# Metabolic Rate of Goldfish (*Carassius auratus*) in the Face of Common Aquaculture Challenges

**DOI:** 10.3390/biology13100804

**Published:** 2024-10-09

**Authors:** Lisbeth Herrera-Castillo, Germán Vallejo-Palma, Nuria Saiz, Abel Sánchez-Jiménez, Esther Isorna, Ignacio Ruiz-Jarabo, Nuria de Pedro

**Affiliations:** 1 Department of Genetics, Physiology and Microbiology, Faculty of Biological Sciences, University Complutense of Madrid, 28040 Madrid, Spain; lisbethh@ucm.es (L.H.-C.); germanva@ucm.es (G.V.-P.); nursaiz@ucm.es (N.S.); eisornaa@ucm.es (E.I.); ignacio.ruizjarabo@csic.es (I.R.-J.); 2 Department of Biodiversity, Ecology and Evolution, Faculty of Biological Sciences, University Complutense of Madrid, 28040 Madrid, Spain; abelsanc@ucm.es; 3 Department of Aquaculture, Institute of Marine Sciences of Andalusia (ICMAN-CSIC), 11519 Puerto Real, Cadiz, Spain

**Keywords:** 2-phenoxyethanol, clove oil, fish, MS-222, oxygen consumption, respirometry, stress, temperature, welfare

## Abstract

**Simple Summary:**

This study investigated the response of goldfish (*Carassius auratus*) to different management conditions by assessing their oxygen consumption using an intermittent respirometry system, which serves as an indicator of metabolic rate. Goldfish consumed more oxygen during the day than at night, agreeing with the diurnal pattern of this teleost. Feeding to satiety increased their oxygen consumption by 35%, peaking 3 h after eating and returning to the normal routine values after 7 h. Handling the fish for five minutes caused a 140% increase in oxygen use, which returned to normal after 2.5 h. Increasing the water temperature gradually to 30 °C resulted in a 200% increase in oxygen consumption 2.5 h after the start of the temperature increase. Common aquaculture anesthetics also affected oxygen use for at least 4 h post-recovery. These findings suggest that metabolic rate is a valuable indicator of how goldfish respond to different practices that might increase their energy expenditure and stress. Therefore, intermittent respirometry, which measures oxygen consumption over time, is recommended to help understand and improve fish welfare in aquaculture.

**Abstract:**

This study examined the metabolic rate (MO_2_, oxygen consumption) of goldfish (*Carassius auratus*) under normal management conditions in aquaculture. Using an intermittent respirometry system, we assessed daily variations and the effects of feeding, handling, temperature increase, and anesthetics. MO_2_ exhibited a daily rhythm, with higher values during day. Feeding to satiety produced a 35% increase in MO_2_ compared to fasted animals, with a maximum peak after 3 h and returning to baseline after 7 h. Handling stress (5 min) produced a 140% MO_2_ peak (from 180 to 252 mg O_2_ kg^−1^ h^−1^), returning to the routine MO_2_ after 2.5 h. An increase in water temperature (+0.1 °C min^−1^) up to 30 °C caused MO_2_ to peak at 200% after 2.5 h from the start of the temperature increase. The use of common anesthetics in aquaculture (MS-222, 2-phenoxyethanol and clove oil in deep anesthesia concentration) affects MO_2_ during the first few minutes after anesthetic recovery, but also during the following 4 h. It can be concluded that the metabolic rate is a good indicator of the goldfish’s response to aquaculture practices involving energy expenditure and stress. Thus, intermittent respirometry is a valuable non-invasive tool for understanding and improving fish welfare in aquaculture.

## 1. Introduction

Aquaculture, in accordance with the principles of blue economy, is presented as the most sustainable alternative for the production of animal proteins of aquatic origin [1]. The current growth of aquaculture is exponential, with the industry playing a significant role in the global food supply, primarily through the production of fish. This development is linked to a series of problems that need to be solved. Some of these are unique to a given species under cultivation, while others are more general, such as improving growth rates, minimizing the appearance of diseases, and optimizing economic returns [2]. The aforementioned aims are all linked to ensuring animal welfare, which is of great concern in the context of modern societies [3]. Thus, the advancement of aquaculture is contingent upon the provision of optimal fish welfare conditions [4]. This concept is inherently complex, and the necessity to quantify it further complicates the matter [5]. Consequently, approaches based on physiological analysis of stress responses have been proposed as a means of defining and measuring animal welfare [6]. These physiological responses aim to meet the energy demands required to cope with stress, producing important alterations in metabolism.

Energy management is, in general, well studied and presents common aspects in all taxa [7,8,9,10], thus allowing comparisons between different species. However, the study of changes in the metabolic state of animals typically requires prolonged periods of observation (e.g., measuring growth rates or changes in condition factor) or the use of invasive methodologies (e.g., studying energy reserves or the state of intermediary metabolic pathways). Alternatively, the overall performance and physiological state of the fish can be measured through indirect methodologies, such as oxygen consumption [11]. In this context, the utilization of intermittent respirometry to assess metabolic rate in fish represents a highly effective and cutting-edge approach [12]. This methodology enables the real-time acquisition of precise information on the physiological responses of the organism through non-invasive methods. It allows for the assessment of an organism’s capacity for acclimatization to varying environmental conditions, as well as for the evaluation of secondary responses to acute and punctual changes [13,14].

Stress responses in fish, as in vertebrates in general, can be classified into three categories: primary, secondary, and tertiary [15]. The primary category encompasses responses related to hormone production, while the secondary category covers physiological responses to these hormones [16]. These include an increase in the metabolic rate to cope with the changes experienced by the animal, mobilization of energy resources, and increased oxygen consumption. Tertiary responses, which occur over the medium to long term, are reflected in lower animal growth rates and immunosuppression, with greater sensitivity to infections and reproductive impairments, among other problems [17]. In light of this, it becomes evident that identifying the early responses to stress in aquaculture is of major importance, with the objective of preventing significant issues from arising. This would enable prompt intervention and the maintenance of optimal conditions for the animals, which is crucial for their well-being. Consequently, an examination of the metabolic rate of the animals through the measurement of oxygen consumption appears to be an effective approach for determining the potential for stress, as it represents a non-invasive, rapid, and efficacious methodology.

Common husbandry practices employed in aquaculture may have a deleterious effect on the physiology of fish [18]. However, the extent to which these practices may influence the animals’ physiology is mostly unknown due to species-specific peculiarities [3]. It is therefore essential to gain a better understanding of the effects of these practices in order to prevent them or to implement corrective management methodologies, as well as to make uniform analytical methodologies for all fish species, which are fundamental to the advancement of aquaculture. A number of maintenance protocols would require evaluation, including those pertaining to the transfer of fish between tanks, changes in water temperature, the impact of feeding or anesthesia, and others. Thus, some studies have indicated that the process of capturing fish is inherently stressful [19,20] and can be minimized if management protocols are improved. Temperature exerts a direct influence on the metabolic rates of fish, which are ectotherm animals [11,21], including their digestive processes, as it modifies oxygen consumption [22]. In fact, temperature is the most important factor affecting metabolic rate in ectotherms, due to its effect on the kinetic energy of cellular components [23]. Feeding time is crucial for fish well-being [24], and understanding of the postprandial wave, which elevates oxygen consumption and diverts metabolic energy to digestive processes [25], may also prove beneficial in the context of fish management. The study of anesthetics to manipulate fish and improve their welfare is a growing field. Numerous anesthetic compounds are available, but to date none have been described as being universal, due to species-specific and age differences [26]. The existence of day–night differences in anesthesia and/or recovery time [27,28] should also be taken into consideration, as well as the fact that the effect of an acute stressor may depend on the time of day [29]. This may impact the daily variations in the metabolic rate of fish, potentially being relevant for day and night maintenance conditions in aquaculture.

The aim of this study is to analyze oxygen consumption as a non-invasive tool to evaluate the effect of different management processes commonly occurring in aquaculture. This approach will facilitate the assessment of daily variations in metabolic rate, along with the impact of feeding, handling, temperature changes, and the use of anesthetics. The goldfish (*Carassius auratus*), a freshwater fish commonly employed in research [30], will serve as the study model. This Cyprinid species is representative of the most widely cultured and studied fish family in the world [1].

## 2. Materials and Methods

### 2.1. Animal Model and Housing

Goldfish juveniles were obtained from commercial sources (ICA S.A., Madrid, Spain) and housed in the animal husbandry facility (Biological Sciences Faculty, University Complutense of Madrid, Spain). Fish were maintained in groups of 4 animals in 60 L tanks in a recirculated water circuit with a biological filter. The water temperature was maintained at 21.5 ± 1.0 °C (mean ± standard deviation), and the photoperiod was 12L:12D (lights on at 08:00 h, *Zeitgeber* Time 0 or ZT0). Fish were fed once a day at 10:00 h (ZT2) with 1.5% body weight (bw) with commercial dry pellets (32.1% crude protein, 5% crude fat, 2.6% crude fiber, 5.2% crude ash; Sera Pond BioGranulat, Heinsberg, Germany) with automated feeders. All animals were acclimated for more than 14 days to described conditions before the experiments. The housing conditions for the fish in all experiments are as described in this section, unless stated otherwise in the experimental design.

Fish were anesthetized with 0.14 g L^−1^ MS-222 (tricaine methane sulfonate, Sigma-Aldrich, St. louis, MO, USA) buffered with sodium bicarbonate and individually labelled by means of sub-dermal ink dots (Eternal Ink, Barnsley, UK) to be able to identify individual fish. This study was performed in accordance with the Guidelines of the European Union (2010/63/UE) and the Spanish legislation (RD 53/2013) for the use of laboratory animals and was approved by the Animal Experimentation Committee of Complutense University and the Community of Madrid (PROEX 317.7/23). This study did not involve endangered or protected species.

### 2.2. Experimental Designs

#### 2.2.1. Daily Rhythm of the Locomotor Activity and Metabolic Rate of *C. auratus*

Locomotor activity of 8 goldfish (17.1 ± 3.1 g bw, mean ± S.E.M. or standard error of the mean) was registered for 9 days to confirm fish acclimation to the photoperiod and the feeding time. After 48 h of fasting, fish were transferred to respirometry chambers (10:00 h) and the metabolic rate was measured for 48 h.

#### 2.2.2. Effect of Feeding

Oxygen consumption was measured in goldfish (24.5 ± 5.4 g bw, *n* = 8) under two conditions: (a) 48 h of fasting and (b) 48 h of fasting followed by feeding for 30 min at satiety (3% bw) at the acclimation feeding time (10:00 h). Measurements were initiated immediately upon the transfer of the fish to respirometry chambers (10:30 h) and continued for a period of 24 h to evaluate the postprandial cycle until complete digestion of food, as described in other Cypriniformes [31]. Both conditions (fasting and refeeding) were tested on the same individuals one week apart.

#### 2.2.3. Effect of Acute Stress

Goldfish (25.2 ± 1.9 g bw, *n* = 8) were transferred to respirometry chambers at 17:00 h and kept overnight to eliminate the effect of handling. At 10:30 h (in 24 h fasting conditions) acute stress was caused by chasing the fish into the chambers for 5 min, which has already been tested in other teleosts as a valid method to induce acute stress responses [19,32]. The oxygen consumption of the fish was analyzed during the 6 h following their exposure to acute stress. This period of time was deemed sufficient to allow for the observation of physiological recovery in other teleosts [33].

#### 2.2.4. Effect of Temperature

Goldfish (20.9 ± 0.9 g bw, *n* = 8) were transferred to respirometry chambers as described in the previous experiment in Section 2.2.3. At 10:30 h (in 24 h fasting conditions) the temperature was gradually increased (0.1 °C min^−1^) from 21 °C to 30 °C. The temperature change process was conducted with the introduction of a water heater into the general basin, which also housed the respirometry chambers. Furthermore, the system incorporated a water cooler to ensure precise temperature regulation. The system was equipped with water pumps that facilitated rapid and effective mixing. We selected 30 °C as the temperature *C. auratus* can acutely tolerate without experiencing any adverse effects on its behavior, while the gradual temperature increase was chosen as one-third of that tested to calculate the critical thermal maximum (Ct max) in this species [34]. Metabolic rate was measured for 5 h after the temperature increase. The thermal coefficient Q10 was calculated as previously described [35].

#### 2.2.5. Effect of Different Anesthetics

Goldfish (22.2 ± 6.8 g bw) were divided in four groups (*n* = 8–10 per group), and all animals were maintained in a 24 h fasting state: control group (fish were directly transferred to respirometry chambers) and three groups of anesthetized fish with 2-phenoxyethanol (0.5 mL L^−1^), clove oil (100 µL L^−1^), or buffered tricaine methane–sulfonate (MS222, 0.14 g L^−1^) (Sigma Chemical, Madrid, Spain). The protocol for the anesthetized fish groups was as follows: Fish were placed in fresh, filtered water with the anesthetic for 5 min. The selected doses were sufficient to induce deep anesthesia, characterized by a total loss of equilibrium, reflexes, and locomotor activity in this and other teleost species [36,37,38,39]. This state was maintained while the animal was placed in the respirometry system. Fish were maintained in the respirometry chambers for 4 h, allowing them to completely recover from anesthesia while oxygen consumption was recorded.

### 2.3. Determination of Metabolic Rate

Oxygen consumption rate (MO_2_) of individual goldfish was measured by intermittent flow respirometry (Loligo Systems, Viborg, Denmark). Each animal was placed individually in a 362.29 mL respirometer chamber under the same environmental conditions as their acclimation tanks (except for temperature in the experiment in Section 2.2.4, as explained above) with water flowing inside the chambers (300 L h^−1^). Oxygen optode sensors and a temperature probe were connected to a Witrox-4 module and the AutoResp™ software (version 2.3.0) was employed for the data acquisition (Loligo Systems, Viborg, Denmark). The system included wash and recirculation water pumps for each chamber and a temperature control module.

Optimization of the respirometry system was carried out following the criteria and the recommendations of previous studies [12,40]. One complete oxygen consumption measurement cycle consisted of an open-system flush period (75–79 s), and a closed system with a waiting period (1–10 s) and a measurement period (75–150 s). The washing, waiting, and measurement times were adapted to each experimental condition to minimize the cycle time. The oxygen saturation in the wash tank was maintained through the utilization of air diffusion stones, which ensured a constant and stable supply of oxygen. The duration of the measurement phase ensured the oxygen saturation levels were above 80%, avoiding hypoxic stress, and the coefficient of determination (R^2^) associated with each MO_2_ measurement was >0.95. The concentration of oxygen in the empty chamber was measured for 25 min before and after each experiment to estimate background respiration (i.e., microbial respiration). Background metabolism in our respirometry system was <1% of total measured metabolism, which is considered insignificant [40].

Before each experiment, the chambers were cleaned and disinfected, and the oxygen sensors were calibrated using 100% (with aeration systems) and 0% (with nitrogen gas) oxygen-saturated water. Each fish was weighed and transferred into a respirometry chamber with minimum chasing to reduce the initial stress [41]. Then, the acquisition started, and the experiment was controlled remotely to prevent external disturbances. Using the automatic values taken during each measuring phase, the program calculates the mass-specific oxygen rate (mg O_2_ kg^−1^ h^−1^) using the equation MO_2_ = V∙M^−1^∙(d[O_2_])/dt, where V is the respirometer volume minus the volume of the experimental animal (L), M is the body weight of the fish introduced in the experiment (kg), and d[O_2_]/dt is the slope of the linear decrease in oxygen content during the time the chamber is closed.

### 2.4. Locomotor Activity Recording

The locomotor activity of the fish was recorded for 9 days as described [42], with 4 infrared photocells (E3S-AD12, OMRON Corporation, Kyoto, Japan) stuck to the aquaria walls. Tanks were covered with opaque paper to minimize visual contact between tanks. All photocells were connected to an actimeter controlled by data-acquiring software (Adq16, a non-commercial software specifically developed for this purpose by Micronec, Madrid, Spain). The number of light beam interruptions was automatically registered every 10 min, and all data were analyzed using EL TEMPS^®^ (Prof. Antoni Díez Noguera, University of Barcelona, Spain) to obtain profiles of averaged daily rhythms, actograms, and periodograms.

### 2.5. Statistical Analysis

Data are expressed as mean ± S.E.M. The normality and homoscedasticity of the data were confirmed by the Shapiro–Wilk and Levene tests, and when necessary, data were transformed to a logarithmic scale. A two-way ANOVA was conducted to compare the possible differences in the metabolic rate of the two days and their scotophase and photophase, followed of a Holm–Sidak multiple comparisons test. The paired Student *t*-test was used to compare the oxygen consumptions between fasted fish and fed fish. Significance was considered at *p* < 0.05. All these analyses were conducted with Sigmaplot^®^ software (version 12.0).

The existence of significant periods in daily locomotor activity was analyzed by constructing chi-square periodograms with a significance level set at 0.05 (EL TEMPS^®^, University of Barcelona, Spain). The Cosinor Online application [43] was used to determine if there was a daily significant rhythm in the metabolic rate. Data were adjusted to a sinusoidal function: Y = M + A × cos (t × π/12 − Φ), with M being the mesor (mean values of the rhythm), A the amplitude (difference between the mesor and the maximum value), t the time, and Φ the acrophase (time where the maximum value is reached). The significance of the Cosinor analysis was defined by the test of amplitude zero.

To study the influence of temperature, acute stress, and anesthetics on the metabolic rate over time, the existence of breakpoints (i.e., changes in the slope) was initially evaluated through piecewise regression (package segmented) [44]. Subsequently, each data segment (between breakpoints) was analyzed individually using linear mixed-effects models with restricted maximum likelihood (REML) estimation, with each fish serving as the grouping factor (package lme4) [45]. In all cases, a tentative complete model that included all factors and random effects was used. Then, non-significant factors, both random and fixed, were removed following a backward stepwise approach until a final model with all significant factors was reached. Time was included as factor of variance in all three studies. In the case of temperature effect, both time and temperature were included as fixed and random effects, while to compare anesthetics, a categorical variable identifying each group was included in the model. Comparative analyses of mean consumption at specific points in time and time trends between anesthetics were conducted using the emmeans package [46], using the Tukey test for multiple comparisons with the FDR *p*-value adjustment method. All these analyses were carried out with R (version 4.3.3.) [47] and RStudio (version 2024.4.2.764) [48] software.

## 3. Results

### 3.1. Daily Rhythm of the Locomotor Activity and Metabolic Rate of C. auratus

**Figure 1:**
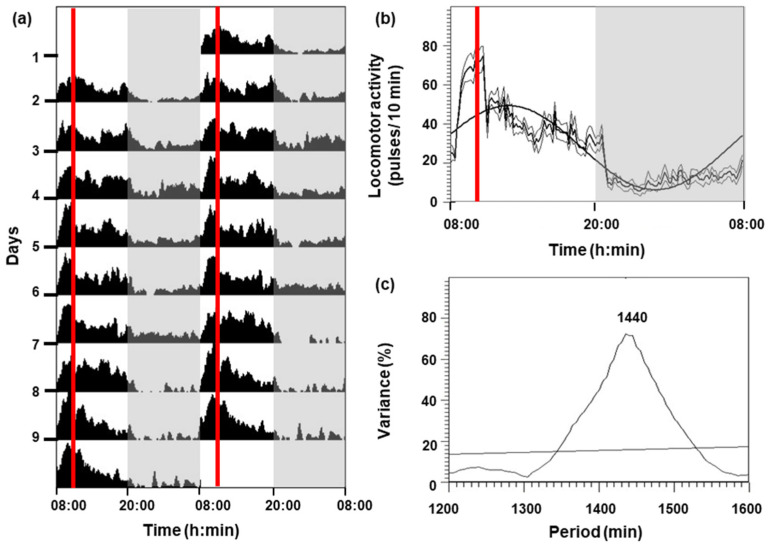
Locomotor activity of goldfish for 9 days. (**a**) The actogram is represented as double-plotted (48 h time scale) for better visualization. (**b**) Average waveform: mean (bold black line), standard deviation (gray lines), and periodic sinusoidal function wave. The white areas correspond to the light phase (08:00–20:00 h) and the shaded areas correspond to the dark phase (20:00–08:00 h). The red vertical lines indicate the feeding time (10:00 h). (**c**) The periodogram represents the % variance versus time. The horizontal line reflects the significance threshold (*p* < 0.05) and the number above the observed peak indicates the value of the period in minutes.

The locomotor activity of *C. auratus* for 9 days is shown in Figure 1. Goldfish under a 12L:12D photoperiod and feeding at 10:00 h had higher activity during the photophase (08:00–20:00 h) than in the scotophase (20:00–08:00 h), showing an increment in activity 2 h before the feeding time (Figure 1a,b). A significant diurnal rhythm (*p* < 0.05) was displayed (Figure 1c), with the following parameters: mesor (27.83 pulses per 10 min), amplitude (21.5 pulses per 10 min), and acrophase (12:42 h).

**Figure 2:**
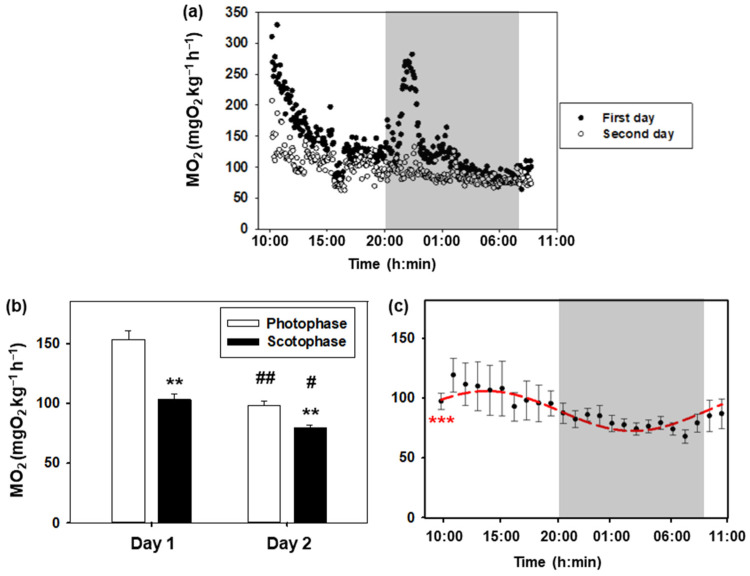
Daily oxygen consumption of goldfish. (**a**) The 24 h profile of the metabolic rate of 8 goldfish during the first day (black dots) and the second day (white dots). The white areas correspond to the light phase (08:00–20:00 h), while the shaded areas correspond to the dark phase (20:00–08:00 h). (**b**) Oxygen consumption during photophase and scotophase on days 1 and 2. Data are represented as mean + S.E.M. (*n* = 8). Two-way ANOVA, Holm–Sidak test: ** *p* < 0.001 for photophase versus scotophase on the same day; # *p* < 0.05; ## *p* < 0.001 for the same phase of the day comparing days 1 and 2. (**c**) Metabolic rate (mean ± S.E.M.) of *C. auratus* on the second day (data are grouped by hours). As the Cosinor analysis yielded significant results (Zero amplitude test: *** *p* < 0.001), the sinusoidal periodic function is represented as a red curve.

Daily variations in the oxygen consumption rate of goldfish are represented in Figure 2. The daily profile of the first and second day is similar, with higher values during the day than during the night (Figure 2a,b). Notably, the values during the first 24 h are higher than those recorded during the second day, with these differences being more significant during photophase (*p* < 0.001) than during scotophase (*p* < 0.05). In addition, there was an increase associated with turning off the lights on the first day, which was not observed on the second day. The Cosinor analysis of metabolic rate values obtained during the second day is shown in Figure 2c. A significant 24 h rhythm (*p* < 0.001) was observed with the following parameters: mesor (90.5 mg O_2_ kg^−1^ h^−1^), amplitude (16.9 mg O_2_ kg^−1^ h^−1^), and acrophase (13:12 h).

### 3.2. Effect of Feeding

**Figure 3:**
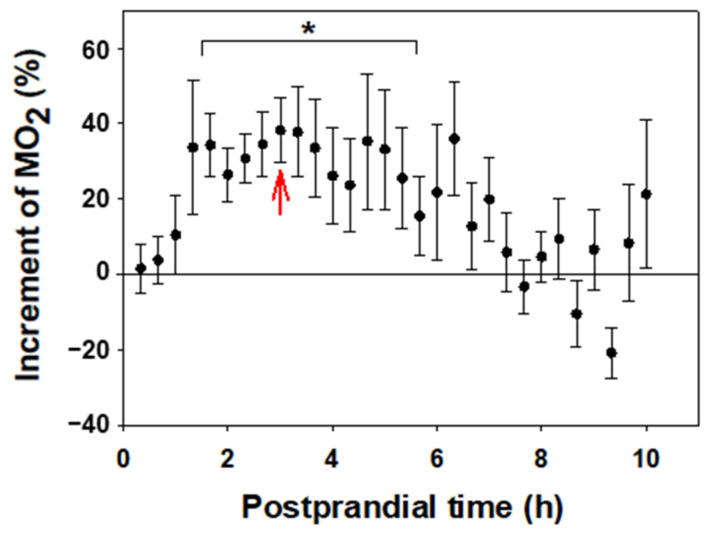
Effect of feeding on the metabolic rate of *C. auratus*. The % variation in MO_2_ of fed fish with respect to the fasting group is represented by the mean ± S.E.M (*n* = 8 per group). Data are grouped by 10 min intervals. The points at which there are significant differences are indicated with a bracket and * (Paired Student *t*-test, *p* < 0.05), and the red arrow indicates the time of maximum difference.

Oxygen consumption in recently fed fish was higher than in the same fish fasted for 48 h (Figure 3). A significant increase (*p* < 0.05) was found from 1 h and 40 min to 5 h and 40 min after food intake, with the maximum value 3 h after feeding (Δ = 35%). The observed differences were not statistically significant from 6 h post-feeding, returning to baseline values by 7 h.

### 3.3. Effect of Acute Stress

**Figure 4:**
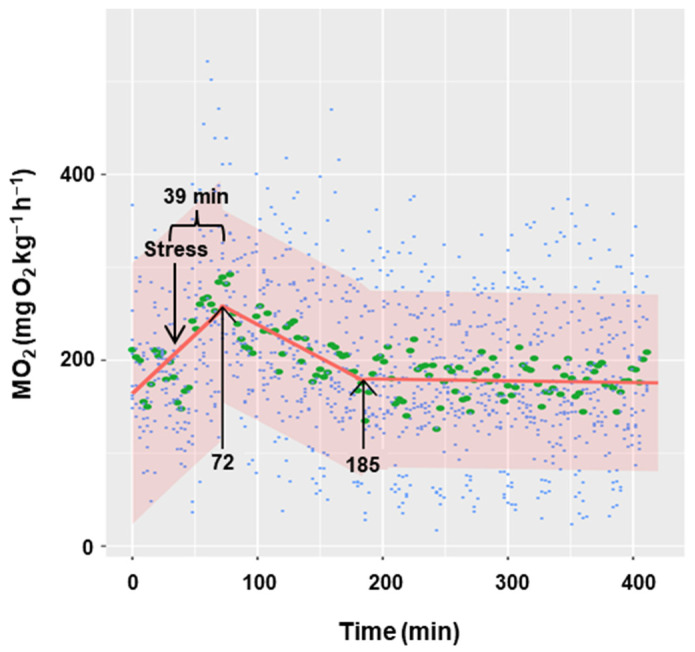
Effect of acute stress on the metabolic rate of *C. auratus*. The blue points represent the raw data of 8 fish, the green points correspond to the mean of all values at each time, and the red line represents the regression line. Shaded regions correspond to 95% confidence intervals. The first arrow indicates the time of the maximum MO_2_ and the second arrow the time when stabilization is reached.

Acute stress (handling for 5 min) significantly increased oxygen consumption at a rate of 1.26 units per minute (*p* < 0.001), reaching the maximum (252.02 mg O_2_ kg^−1^ h^−1^) 39 min after the handling (Figure 4). Subsequently, for the next 113 min (from 72 to 185 min), oxygen consumption began to decrease at a rate of −0.72 units per minute (*p* < 0.001). From this moment onwards, oxygen consumption remained constant (slope = −0.0184), with an estimated basal consumption averaging 180 mg O_2_ kg^−1^ h^−1^.

### 3.4. Effect of Temperature

**Figure 5:**
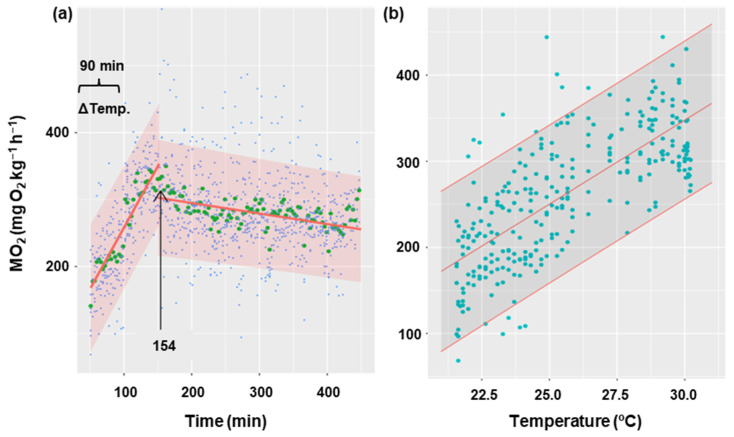
Effect of temperature increase on metabolic rate in *C. auratus*. (**a**) The blue points represent the raw data of 8 fish at each time, the green points correspond to the mean of these fish at each time, and the red line represents the fit of the data to a lineal regression. The arrow indicates the time at which the linearity break occurs (154 min). (**b**) The relationship between temperature and oxygen consumption during the first phase (154 min), with the raw data (blue) of 8 fish at each temperature represented with their fitted values (red). Shaded regions correspond to 95% confidence intervals.

Figure 5 shows the oxygen consumption rate in goldfish during the gradual increase in temperature from 21 °C to 30 °C for 90 min, and 5 h after the increase. It has been demonstrated that an increase in temperature results in a corresponding increase in oxygen consumption (*p* < 0.001), with a rate of 19 MO_2_ units per degree Celsius of temperature (Figure 5b). Oxygen consumption showed a gradual increase until it reached a maximum (352 mg O_2_ kg^−1^ h^−1^) at minute 154 from the start of recording oxygen consumption. At this point, MO_2_ began to decrease (Figure 5a) significantly (*p* < 0.05) at a rate of 0.16 units per minute. This decline appeared to stabilize, although the duration of the experiment may not have been sufficiently long enough to detect the potential for an asymptote. The thermal coefficient (Q10 = 3.06 ± 0.38) was calculated based on the initial and final temperatures for each animal (21 and 30 °C, respectively), as well as the MO_2_ registered 30 min before the temperature increase (averaging 112 ± 5 mg O_2_ kg^−1^ h^−1^) and the final oxygen consumption (averaging 278 ± 2 mg O_2_ kg^−1^ h^−1^).

### 3.5. Effect of Different Anesthetics

**Figure 6:**
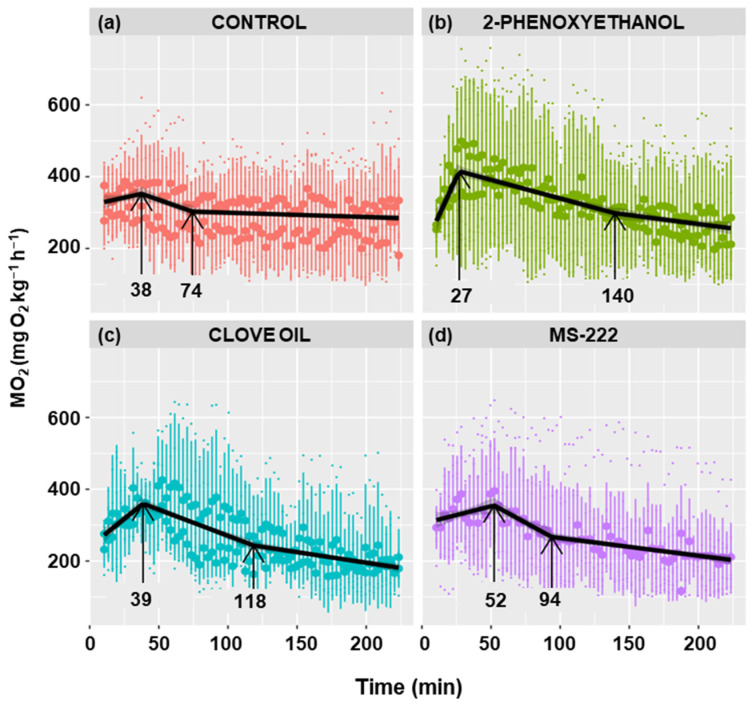
Effect of different anesthetics on the metabolic rate of *C. auratus*. The black line represents the fit of the data to a line regression. Shaded regions correspond to 95% confidence intervals. The first arrow indicates the time of the maximum MO_2_ and the second arrow the time when stabilization is reached.

Figure 6 shows the metabolic rate profiles of anesthetized and non-anesthetized goldfish. A similar profile is observed in all cases, characterized by a positive slope in oxygen consumption, followed by an inflection point at which the maximum is achieved and the slope becomes negative. The maximum was reached at 38 min in the control group, at 27 min in the 2-phenoxyethanol group, at 39 min in the clove oil group, and at 52 min in the MS-222 group, although there were no statistically significant differences.

The maximum MO_2_ was similar in all four experimental groups, although fish anesthetized with 2-phenoxyethanol had a slightly higher mean O_2_ consumption (Table 1). The 2-phenoxyethanol decreased the time to peak O_2_ consumption and increased the maximum consumption value, resulting in a significantly (*p* < 0.01) higher rate of increase in consumption (slope) than in the other groups (Table 1). Clove oil caused a higher slope as well; however, it was only statistically significant compared to MS-222 (*p* < 0.01), being nearly significant when compared with the control group (*p* < 0.07).

The rate of decrease in consumption was statistically higher in the control (*p* < 0.05) and MS-222 (*p* < 0.001) groups compared to the other groups (Table 1). The time to reach the baseline consumption is defined as the stabilization time. Even though there were no significant differences, it was observed that the control group had the lowest stabilization time (74 min), while the highest value was observed in the 2-phenoxyethanol group (140 min) (Table 1). The anesthetics had lower baseline consumption than the control group, being significantly different than the clove oil (*p* < 0.05) and MS-222 (*p* < 0.01) groups and close to significance (*p* < 0.07) when compared to the 2-phenoxyethanol group (Table 1).

## 4. Discussion

The present study demonstrates that maintenance routines can result in considerable alterations in oxygen consumption rates in fish. Moreover, we measured the recovery times following different challenges (stress, anesthetics, temperature increase), which is essential to preventing the combined impact of multiple stressors on the same animal simultaneously. Thus, by using intermittent respirometry we can determine husbandry limits with greater precision, without invasive procedures.

### 4.1. Daily Variations

Oxygen consumption depends on the time of day, since higher values were observed during photophase in goldfish, with a maximum at midday (ZT5, 12:42 h). This aligns with daily rhythm in locomotor activity, coinciding with previous studies in this same species [42] and confirming their diurnal pattern [49]. Since the animals were fasted for 48 h, the effects of specific dynamic action (SDA) can be ruled out, attributing the observed changes only to the time of day. These findings suggest that the daily rhythm observed in the goldfish’s metabolic rate can be synchronized with the light/dark cycle, as demonstrated in other teleost species under similar conditions. A daily rhythm was described, with higher oxygen consumption during the day in diurnal species like the Nile tilapia (*Oreochromis niloticus*; [50]) and the Mexican tetra fish (*Astyanax mexicanus*; [51]), or during the night in nocturnal species like the Senegalese sole (*Solea senegalensis*; [52]) and epaulette shark (*Hemiscyllium ocellatum*; [53]). These daily rhythms persist in the absence of environmental synchronizers, during fasting, and in constant darkness or light conditions [50,52,53,54], suggesting they are circadian rhythms. The metabolic rate in mammals also displays daily rhythmicity, which seems to be controlled by the circadian clock either directly or indirectly through clock-regulated rhythms (rest–active), being widely conserved among vertebrates [55].

It is noteworthy that the metabolic rate observed in the present study varied according to the duration of acclimation to the respirometry chambers, as illustrated in the 48 h trial, causing an initial period of elevated oxygen consumption, as observed in goldfish during the first few hours. This phenomenon was previously described in ballan wrasse (*Labrus bergylta*) in a 5-day acclimation period [56] and *A. mexicanus* in a 7-day acclimation period [51]. The authors of both studies proposed that fish would require at least 24 h to acclimate to the metabolic chamber, as is also the case of goldfish. The MO_2_ measured in our experimental conditions was closer to the routine metabolic rate, as fish can only show some minor activity (swimming and maintaining position) inside the respirometry chambers [41]. Thus, the lowest MO_2_ recorded during the second night (76.95 mg O_2_ kg^−1^ h^−1^) was close to a standard metabolic rate, as the fish would be fully acclimated with the lowest levels of locomotor activity, and in a fasting state, unaffected by SDA [41]. Therefore, it is essential to carry out the analyses of the same experiment at the same time of day to be able to compare the results of oxygen consumption. Furthermore, in the present work, it was observed that there was a significant increase in the metabolic rate after the lights were switched off on the first day, which also occurred the second day, although in a more attenuated way. The abrupt change in lighting, without a gradual light-to-dark transition, may induce acute stress in fish, triggering the release of catecholamines and cortisol. This hormonal response likely contributes to an elevated oxygen consumption as a physiological reaction to stress. It has been suggested for some years that there may be welfare problems in fish due to rapid light transitions [4], so dimming options to graduate the onset and offset of lighting would be desirable. These results also show that after 24 h in the chamber, animals can cope better with stressors. Although there is a substantial body of literature that recommends acclimating fish for a couple of hours to respirometry chambers prior to conducting metabolic rate studies [12,41,57], the present results indicate that this time could be insufficient. It is also essential to consider other factors. Consequently, the design of future experiments to evaluate the oxygen consumption of animals should include, in addition to the classical variables (physicochemical parameters and chamber volume, among others) or the gregarious nature of individually confined animals, the potential for a measurement artifact resulting from the length of time the animal has been in the respirometry chamber. According to these premises, it seems prudent to establish a criterion of 12–24 h of acclimation to confinement in order to obtain metabolic rate data starting from baseline levels of oxygen consumption, without a significant bias due to the current methodological approaches.

### 4.2. Feeding

According to our study, the specific dynamic action (SDA) of the food is reflected in an increase in oxygen consumption of the fish lasting about 7 h after ingestion. This process, peaking at 3 h, coincides with previous studies conducted on this species [58]. Digestion represents a constraint on the energy available for other physiological processes, and may be deleterious to the animal’s ability to cope with situations in which it is required to perform activities that necessitate, e.g., swimming, as described in some cyprinid species [58]. As SDA depends on environmental temperature [58], it would be of interest to test the duration and amplitude of the oxygen consumption increase due to digestion in *C. auratus* and other aquaculture-farmed species at different temperatures. This will enable the determination of the minimum time necessary to wait after feeding in order to perform normal farming tasks without adverse impact on the fish. The age of the fish must also be taken into account, as their digestive processes may vary with time [59]. Moreover, it is relevant to be aware of the digestion times and to adhere to them, as feeding synchronizes biological clocks in goldfish [60]. Thus, respirometry has demonstrated its suitability as a methodology for optimizing feeding times and aquaculture management while respecting the time taken for digestive processes.

### 4.3. Acute Stress

The minimum time required for acclimation of the fish after the manipulation necessary to introduce them into the respirometry chambers was not entirely clear and varied depending on the species. Our results in *C. auratus* indicate that oxygen consumption reaches a maximum 39 min after an acute stress situation that emulates the manipulation necessary to introduce the fish into the respirometry chambers or transfer to another tank. Under our experimental conditions, recovery occurred after 152 min (2.5 h). In Atlantic salmon (*Salmo salar*), although it was not calculated, recovery after an acute stress required around 3 to 5 h before reaching an asymptote of oxygen consumption [14]. In previous studies conducted in goldfish, killifish (*Fundulus heteroclitus*), zebrafish (*Danio rerio*), and other Cypriniformes, the minimum acclimation time was not validated and the animals were left in the respirometry chambers for periods ranging from 2 to 24 h before the metabolic rate was measured [13,61,62,63]. It would be prudent to conduct preliminary trials on each species to ascertain the requisite time for the metabolic rate to reach a plateau following the introduction of the animals into the metabolic chambers, as discussed above. On the other hand, as a secondary stress response, changes in metabolic rates are linked to stress hormones (including cortisol in teleost fish). Short periods of air exposure induced increase plasma cortisol levels for at least 2 h in goldfish [64]) and tambaqui (*Colossoma macropomum*; [20]), 4 h in gilthead seabream (*Sparus aurata*; [33]), and 6 h in *S. senegalensis* [65]. It would be advisable to establish a relationship between oxygen consumption and cortisol production in fish after stressful situations. This can be measured in real time, as the hormone can be analyzed in the water that comes out from the respirometry chamber without having to manipulate the animal [66]. This approach would allow for the adjustment of the time required for the animal to acclimate to the respiratory chamber without the necessity of prolonging the period of confinement, thereby improving fish welfare.

### 4.4. Temperature

The results of our study indicate that an acute increase in temperature from 21 to 30 °C over a 90 min period did not result in sub-lethal effects in goldfish. Although the prevailing expectation is that there should be an exponential increase in oxygen consumption in line with increasing temperature [67], the MO_2_ data set is better explained by a linear model with water heating. In order to ensure the welfare of the animals, we avoided reaching the maximum critical thermal limit (Ct max), which is determined by the upper limit at which an animal shows sub-lethal endpoints, such as a loss of equilibrium and stopping breathing [34]. Consequently, an increase of half that necessary to reach the Ct max was selected for *C. auratus* (calculated as 37.9 °C), while the temperature increase was modified at a rate of one-third of the previously described one for this and other species [34,68]. The objective of this temperature increase was to evaluate the responses in metabolic rate in order to extrapolate the methodology to other species, as well as to ascertain the response times and short-term acclimatization capacity. Our calculated Q10 for *C. auratus* metabolic rate (Q10 = 3.06) coincided with that of the elasmobranchs and salmonids, as well as other ectotherms evaluated under similar conditions [23,35,69]. Q10 values of around 2 to 3 are characteristic of typical biological systems in ectotherms. Therefore, it can be assumed that the increase in oxygen consumption observed in our study in response to temperature increase was due to an increase in metabolic enzyme activity and increased energy demand for physiological processes. Additionally, the later study observed that after 6.5 h, the fish did not exhibit the full signs of acclimation, as MO_2_ levels did not stabilize, which is consistent with our results 3 h after reaching 30 °C. Thus, biological imbalances affecting fish homeostasis occur due to short-term thermal stress, requiring the activation of enzyme systems to compensate [70]. It is important to consider that the impact of temperature is influenced by the intensity and duration of exposure, and the rate at which temperature changes occur [71]. Thus, long-term exposure to warmer water will necessitate a complete readaptation of metabolic and general homeostasis regulation systems. In this context, studies conducted with aquaculture species acclimated to different temperatures have demonstrated that the expression of pituitary hormones associated with growth and osmoregulation is altered, as is their systemic distribution [72,73]. These effects have implications for energy management, underscoring the significance of amino acids in this acclimatization process and also for the regulation of the water–ionic balance [74,75]. Certain studies conducted in *F. heteroclitus*, where mitochondrial activity was evaluated, highlighted that small changes in temperature resulted in compensatory increases in mitochondrial performance, while alterations above the thermal acclimatization limits of the species may result in an overload of mitochondrial function, which could potentially lead to mitochondrial collapse [76]. This phenomenon has also been observed in other ectothermic species. For example, in the amphipod *Gammarus insensibilis*, respiratory demand increases with temperature, while oxygen availability and water transport efficiency decrease [77]. With all this information, and as long as the physiological limits of the species are not exceeded, fish are capable of rapidly modifying their enzymatic activity in order to acclimatize to temperature changes in the short term. It is possible that an elevated metabolic rate in warmer waters may have a detrimental impact on the ability to acclimate to additional conditions requiring increased oxygen consumption. Consequently, the utilization of respirometry as a non-invasive methodology proves to be a valuable tool for the monitoring of the physiological thermal limits of fish in aquaculture.

### 4.5. Anesthetics

Although there is a great amount of literature describing the effects of anesthetics in fish, with special emphasis on the drugs employed in the present study [78], there are far fewer studies that evaluate the direct impact of anesthetics on oxygen consumption. Our results in goldfish show that exposure to both clove oil and MS-222 for 5 min induced lower MO_2_ levels than the control group 4 h after the exposure. This is in agreement with preliminary studies conducted in several freshwater fish species, where both anesthetics tended to diminish MO_2_ against a control group [79]. However, neither these two anesthetics nor 2-phenoxyethanol were able to reduce the initial increase in oxygen consumption observed in the control goldfish. This response may be indicative of imbalances during the recovery phase, such as hyperactivity induced by MS-222 in Atlantic sturgeon (*Acipenser oxyrinchus*; [80]), or may even be related to an oxygen deficit due to a ventilation reduction during anesthesia, as was suggested in spotty wrasse (*Notolabrus celidotus*; [81]), although further studies are necessary to fully understand it. Our results highlight that fish exposed to 2-phenoxyethanol showed the highest MO_2_ of all groups, although the maximum peak occurred earlier. This could be associated with a faster recovery process compared to the other anesthetics tested in this study. Although evaluated in crustaceans, 2-phenoxyethanol and clove oil caused disorientation in the spiny lobster (*Sagmariasus verreauxi*) during recovery, enhancing their locomotor activity, increasing their MO_2_ consumption, and requiring longer periods to recover [82]. Something similar occurred in the gammarid *Gammarus pulex*, as MS-222 increased MO_2_ after 33 min of exposure and recovery compared to the control group [83]. These differences in oxygen consumption can be related to changes in locomotion during recovery from anesthesia and are related to slower responses to noxious stimuli after full recovery. According to these studies conducted in crustaceans, the goldfish may face a longer recovery period, with metabolic rate imbalances when anesthetized with MS-222 or clove oil compared to 2-phenoxyethanol. In this sense, eugenol (which is the active principle in clove oil) is more effective than MS-222 for the anesthesia of *C. auratus*, as seen in the transcriptomic changes that occur, although plasma cortisol levels were lower in the MS-222 treated fish [84]. Interestingly, the only anesthetic approved for use in fish intended for human consumption (in some developed countries) is MS-222 due to its rapid metabolization and excretion. However, although it has been proposed that these three compounds may be equally effective in anesthetizing fish, this study demonstrates that respirometry may be a useful tool to elucidate physiological responses that would be obscured by other analytical techniques. Finally, it would be of interest to evaluate the total recovery time of the oxygen consumption rate and to compare it with the locomotor and behavioral responses as an approach to further explore fish welfare [85]. In addition, a deeper examination of the methodology employed would be beneficial in order to ascertain the respiration rates of ex vivo tissues. The latter would facilitate a more comprehensive understanding of the anesthetic mechanisms of the various compounds, thereby providing valuable insights that could inform the selection of anesthetics according to the species or age of the fish in aquaculture.

## 5. Conclusions

In light of the aforementioned considerations, it can be reasonably concluded that the evaluation of whole-body metabolic rate as a means of identifying the physiological limits of fish is a highly useful approach. The utilization of model species, such as the goldfish, has once again been demonstrated to be a fundamental aspect in the development of novel methodologies. This study contributes to the advancement of aquaculture by employing non-invasive techniques to assess the recovery times of farmed fish following standard husbandry procedures. In the future, it would be desirable to establish a correlation between animal oxygen consumption rates and physiological and ethological responses using non-invasive methods. This would facilitate the improvement of fish welfare in aquaculture.

## Figures and Tables

**Table 1:** Effect of different anesthetics on the metabolic rate of *C. auratus*.

MO_2_	Control	2-Phenoxyethanol	Clove Oil	MS-222
Maximum (mg O_2_ kg^−1^ h^−1^)	353 ± 32	415 ± 33 *	359 ± 34	355 ± 32
Increase rate (mg O_2_ kg^−1^ h^−1^ min^−1^)	0.87 ± 0.81	8.34 ± 1.71 **	3.06 ± 0.86	0.99 ± 0.49
Time to maximum (min)	37.5 ± 12.8	27.0 ± 4.8 *	38.6 ± 5.8	52.2 ± 10.5
Decrease rate (mg O_2_ kg^−1^ h^−1^ min^−1^)	0.025 ± 0.006	0.004 ± 0.003 **	0.007 ± 0.004 **	0.042 ± 0.006
Stabilization time (min)	74.3 ± 17.0	139.6 ± 43.2	118.5 ± 22.2	93.9 ± 16.9
Base (mg O_2_ kg^−1^ h^−1^)	288.8 ± 7.8	172.3 ± 129.9	145.7 ± 46.1 *	205.2 ± 22.1 *

Results are shown as mean ± S.E.M. (*n* = 8–10 per group). * *p* < 0.01; ** *p* < 0.001 compared to control group. Statistical differences are also highlighted in bold.

## Data Availability

Data will be available in the DOCTA repository of UCM accessed on 2 of October 2024 (https://hdl.handle.net/20.500.14352/108556).
